# A rare presentation of idiopathic small bowel diaphragm disease – A case report

**DOI:** 10.1016/j.ijscr.2023.108966

**Published:** 2023-10-24

**Authors:** Rajesh R. Ballal, Talha Ahmed, Divya Achutha Ail, Sumith Marian Colaco

**Affiliations:** a Department of General Surgery, Kasturba Medical College, Mangalore, Manipal Academy of Higher Education, Manipal, India; b Department of Pathology, Yenepoya Medical College, Mangalore, India

**Keywords:** Diaphragm disease, Intestinal obstruction, NSAIDs, Small intestine, Case report

## Abstract

**Introduction:**

Diaphragm disease, typically associated with long-term non-steroidal anti-inflammatory drug (NSAID) use, manifests as diaphragm-like small bowel strictures, often resulting in bowel obstruction.

**Case description:**

A 75-year-old male presented with features of recurrent subacute intestinal obstruction, later diagnosed with multiple small bowel strictures via CT imaging. Surgical intervention, including resection and anastomosis, was performed to alleviate the obstruction. Histopathological examination of the resected specimen confirmed diaphragm disease, challenging its traditional association with NSAID use.

**Discussion:**

Diaphragm disease, characterized by mucosal and submucosal diaphragm-like strictures, is typically attributed to NSAID usage. However, this case underscores the possibility of diaphragm disease in the absence of NSAID exposure. Pathological findings supported the presence of diaphragm-like strictures, despite the patient's denial of NSAID use.

**Conclusion:**

This case emphasizes the importance of considering diaphragm disease as a differential diagnosis in patients with intermittent bowel obstruction, even in the absence of NSAID history.

## Highlights

• Diaphragm disease is typically linked to long term NSAID use.
• Presents with diaphragm-like strictures leading to bowel obstruction.
• Crohn's disease or tuberculosis are common differentials.
• Surgical resection of affected segments is the primary treatment with histopathology.
• High index of suspicion is needed to diagnose in the absence of NSAID exposure.

## 1 Introduction

Isolated Small intestinal obstructions are mostly encountered in a postoperative patient due to adhesions, as one of the leading causes [1]. However, diseases like Crohn's, Behçet's, congenital bands, tumours of the small bowel, intussusception, obstructed hernia are few other causes of intestinal obstruction. Clinically, these patients present with symptoms of recurrent small intestine obstructions, unexplained abdominal discomfort, and/or stubborn anaemia brought on by gastrointestinal bleeding [1].

Small-bowel diaphragm, with its first report appearing in the pathologic literature in 1988, makes it a relatively new clinical entity and the usage of non-steroidal anti-inflammatory medicines (NSAIDs) over a long period of time is thought to be the primary cause of this disease [2]. NSAIDs function by preventing the enzyme cyclooxygenase (COX), which in turn affects the production of prostaglandins. However, rather than systemic COX regulation, the specific gut wall changes seen in chronic NSAID users can be attributed to toxicity of the mucosa. Diaphragm like strictures are formed as a result, leading to a usual and rare etiological cause for bowel obstruction.

We describe one such case of a rare occurrence of idiopathic small bowel diaphragm disease in a 75-year-old male patient who had no antecedent history of NSAID usage and who presented with recurrent episodes of subacute intestinal obstruction. This case report has been reported in line with the SCARE Criteria [3].

## 2 Case description

A 75-year-old male presented with abdominal pain and abdominal distension to our surgical outpatient clinic in Mangalore, India. He had similar episodes intermittently for the past 8–10 years which had increased in frequency since the past 1 year. These recurrent episodes were managed conservatively until the current admission. The patient gave history of worsening of pain along with constipation for the last 7 days, which prompted him to seek medical help for the first time. He had no history of vomiting, loss of weight or appetite. On further enquiry about his previous episodes, he explained that he would have similar symptoms of abdominal pain, distension and constipation which would last for 2–3 days, for which he would self-prescribe acetaminophen and bulk laxatives, which would resolve his symptoms. He denied long term use of NSAIDs or any other kind of medications. He gave no history of alternating constipation and diarrhea, bleeding per rectum or tenesmus. He had no history suggestive of stigmata associated with tuberculosis. His past medical or surgical history was not of significance.

On general examination, the patient was tachycardic (109/min), tachypneic (22/min) with a blood pressure of 108/72 mmHg.

On inspection, the abdomen was distended with no surgical scars, sinuses, peristalsis or dilated veins noted. The hernial orifices were free. On palpation, there was no local rise of temperature, however his abdomen was mildly tender. There were no obvious masses that were felt. On percussion, resonant note was heard all over. He had hyperactive bowel sounds on auscultation. There were no signs of liver cell failure or clinical signs of dehydration. Absence of faeces with a roomy rectum was noted on digital rectal examination. Respiratory and Cardiovascular system examinations were within normal limits. There were no abnormalities noted on groin and external genital examination.

At the end of history and clinical assessment, we had reached a probable diagnosis of Intestinal Obstruction with cause to be determined.

## 3 Diagnostic assessment

Routine Baseline Investigations indicated that the patient was mildly anemic with hemoglobin of 11 g/dl and had neutrophilic leukocytosis. His Renal, Liver and Thyroid functions were within normal limits. His blood sugars were within normal range.

A baseline chest X-ray was done which was normal. Erect abdomen X-ray obtained on admission revealed multiple air fluid levels with centrally located dilated bowel loops, suggestive of small bowel obstruction.

**Fig. 1:**
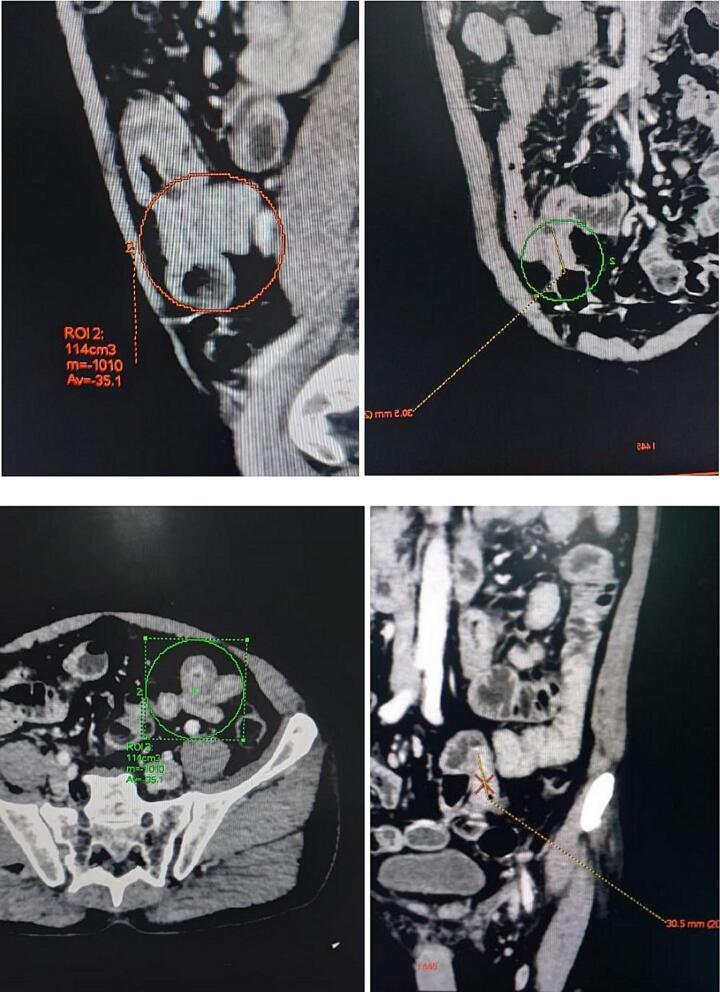
CECT Images showing presence of dilated small bowel loops with multiple strictures in the terminal jejunum and ileum suggestive of small bowel obstruction.

In view of the chronic nature of his history of multiple episodes of subacute intestinal obstruction, a Contrast enhanced CT scan of the abdomen and pelvis was done which revealed the presence of multiple strictures in the small bowel – jejunum and ileum (Fig. 1), leading to small bowel obstruction. There was no evidence of mass lesions, fistula or intrabdominal collection noted. A nasogastric tube was inserted which had a bilious output of 300 ml.

## 4 Therapeutic intervention

In view of the CT report and the presenting clinical condition, the patient was taken up for Exploratory Laparotomy under general anesthesia. The complications of the procedure like resection and anastomoses in case of gangrenous bowel, need for a stoma, reoperation in case of adhesions and future obstruction, wound infection, incisional hernia, hemorrhage was thoroughly conveyed to the patient and written consent was obtained.

The procedure was carried out by two senior gastrointestinal surgeons in our tertiary multidisciplinary hospital who have a collective surgical experience of over 20 years. The procedure was also performed under the care of a specialist anaesthetist and intensivist with an experience of over 19 years.

A midline laparotomy was performed and the abdominal layers were opened to reach the peritoneal cavity. The peritoneum was thoroughly visualized and inspected which revealed dilated jejunal loops. The entire bowel was examined from the D-J flexure till the rectum.

**Fig. 2:**
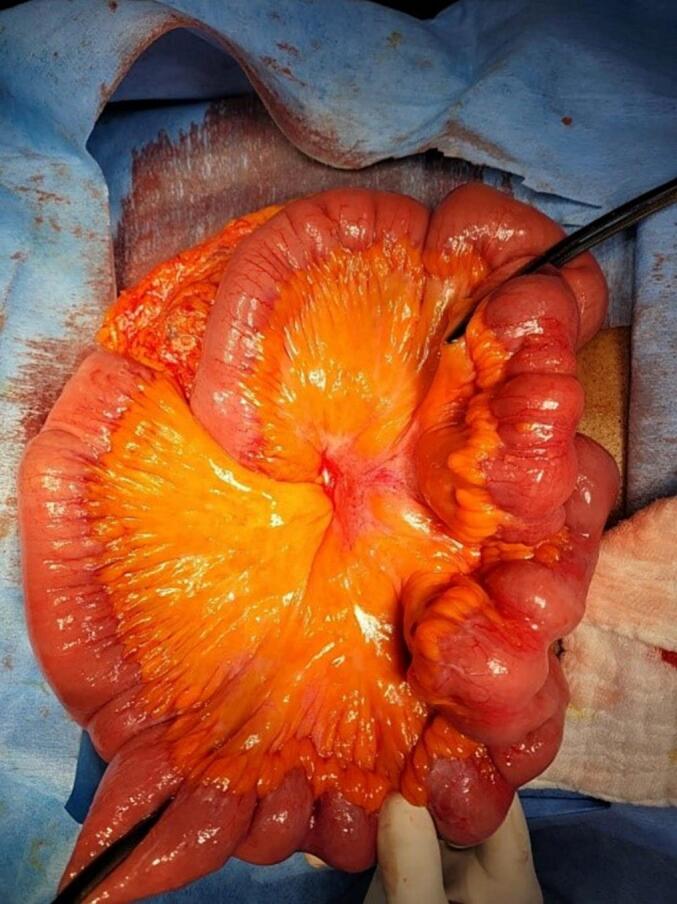
Intra-op image showing strictures in the distal jejunum and proximal ilium of the small bowel with clamps applied proximally and distally indicating level of resection.

The distal jejunal and proximal ileal segment of the small bowel showed the presence of multiple strictures (Fig. 2), approximately 6–7 in number extending for a length of 40 cm with multiple transitional points, about 50 cm from the D-J flexure. The strictures had no evidence of serosal involvement, mesenteric fat encroachment, and had no features of neovascularization. The mesentery along with the mesenteric fat of the involved bowel segments was grossly normal with no evidence of enlarged mesenteric nodes. There were no stigmata suggestive of Inflammatory bowel disease, grossly. There was no evidence of bowel gangrene, masses, internal hernia or enteric fistula noted. There was minimal straw colored inflammatory peritoneal fluid noted, which was sent for culture and sensitivity. In view of the presence of multiple discrete strictures, involving a small segment of 40 cm, a decision to resect the abnormal small bowel segment was taken. This was followed by a single layer hand sewn small bowel anastomoses of the resected ends using 3–0 polyglactin sutures. The mesentery was approximated using 3–0 surgical silk and hemostasis achieved. A single closed suction drain was placed intraperitoneally in the pelvis and the abdomen was closed in layers using non-absorbable monofilament suture (No. 1 loop ethilon) followed by skin closure using staples. The resected small bowel segment was sent for histopathological examination.

## 5 Pathology

On gross examination, resected segment of small bowel measured 40 cm. It showed multiple concentric, almost equidistant strictures formed by fibrous membrane which appeared as exaggerated plica semilunaris. The mucosa over the membrane was ulcerated. Segment between the stricture appeared mildly dilated and appeared to have normal mucosa. Fat stranding was noted in the serosal aspect focally. There was no perforation, mass forming lesion, features of ischemia or gangrenous change of the bowel wall.

**Fig. 3:**
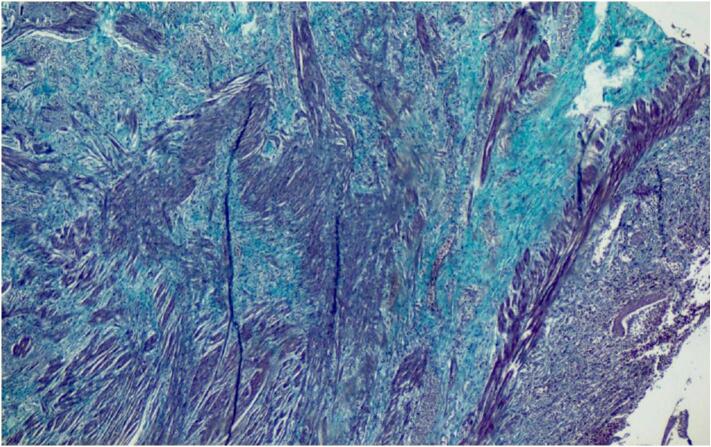
Ulceration restricted to mucosa, increased fibrosis (green) and haphazardly arranged smooth muscle bundles (pink) in submucosa (Masson's trichrome stain, 40×). (For interpretation of the references to colour in this figure legend, the reader is referred to the web version of this article.)

**Fig. 4:**
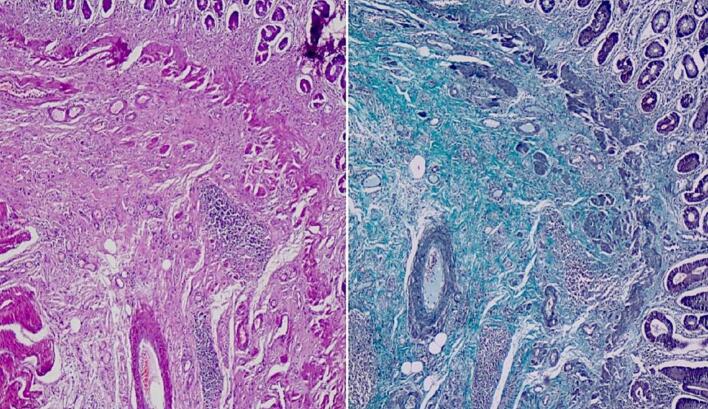
A) Concentric stricture site showing increased fibrosis of the submucosa and thickened muscularis mucosa (Hematoxylin and Eosin, 40×); B) Fibrosis highlighted by Masson's trichrome stain, 40×.

**Fig. 5:**
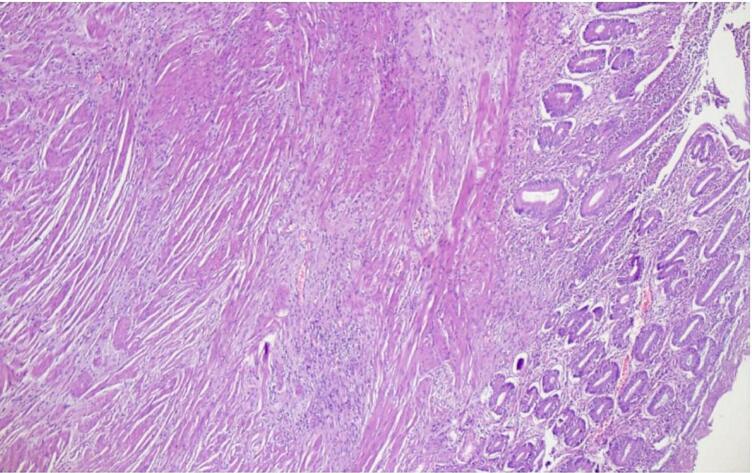
Photomicrography of stricture showing haphazardly arranged smooth muscle fibers and increased fibrosis of submucosa and lack of transmural inflammation (Hematoxylin and Eosin, 40×).

Microscopic examination revealed ulcerated mucosa with acute inflammatory exudates, mildly distorted architecture of crypts with foci of cryptitis and crypt abscess. Underlying submucosa revealed increased fibrosis with interspersed smooth muscle fibers (Fig. 3) and thickened muscularis mucosae forming a band like extension into luminal aspect. (Fig. 4) There is no transmural inflammation. (Fig. 5) There was no presence of granuloma, fissuring, necrosis, dysplasia or neoplasm. Vessels in the subserosa showed mild intimal proliferation of the artery, however there was no thrombosis or vasculitis. 3 Lymph nodes were examined which showed no evidence of caseating granulomas and staining for Tuberculosis was negative. There was no evidence of Crohn's disease.

The impression was of Multiple concentric fibrosis causing membrane like strictures and mucosal ulceration, raising the possibility of Membrane disease.

## 6 Follow-up and outcomes

In view of the above pathology report, the patient was questioned about possible NSAID usage, but he vehemently denied the same. The patient was managed in the post-operative ward. Regular monitoring of his RT and urine output was done. The patient was encouraged to mobilize, which he started from POD1. He was given mechanical DVT prophylaxis and started on active chest physiotherapy exercises. His RT and Foleys were removed on POD 3 and he was started on orals on POD 3. He tolerated orals well and drain was removed on POD 5. His post-operative recovery was uneventful and he was discharged on POD 5. He was tolerating normal diet with regular bowel movements. Patient had come for follow up on POD 30, which showed no evidence of wound dehiscence. Mantoux test was done on discharge, which was negative.

Patient has been on regular 6 monthly follow up with no recurrence of obstructive symptoms.

## 7 Discussion

Diaphragm disease is a rare entity, known to be associated with long-term use of NSAID usage [4] Patients with this disease are asymptomatic till they present with signs of small bowel obstruction. The small bowel obstruction is caused by diaphragm like circumferential membranes resembling plica circularis. Diaphragm like strictures are formed as a result of these local inflammatory effects, which are linked to the formation of reactive oxygen species due to the disruption of membrane phospholipids. A persistent cycle of injury and healing takes place, resulting in the formation of a collagenous scar, leading to intraluminal narrowing, resembling a “drawstring”. This has been hypothesised to cause the development of such strictures [1]. Pathologically, diaphragms are characterized as a disc of tissue that protrudes and narrows the intestinal lumen. Although they might be solitary, diaphragms are frequently numerous and are limited to the mucosa and submucosa. The diaphragm's tip is frequently ulcerated, resulting in a crater-like ulcer bed that can occasionally give the diaphragm a club-like appearance [2].

These diaphragm-like strictures can reduce the size of the lumen to a large extent. They are formed by chaotic, overgrowth of smooth muscle bundles in the muscularis mucosae and fibrosis of the lamina propria during the healing process of small bowel injury or ulcers [5].

Clinically, diaphragm disease is often mistaken as Crohn's disease or tuberculosis due to the presence of multiple strictures. The ulcers in diaphragm disease are limited to the mucosal layer unlike Crohn's disease. In our case, the patient was mildly anemic which is a commonly noted clinical feature in patients with diaphragm disease [6]. Inflammatory bowel disease are characterized histologically by features of chronicity which can be in the form basal plasmacytosis and crypt architectural distortion with respect to mucosal changes. With respect to resection specimens, deep fissuring/serpiginous ulcerations, transmural inflammation, fibrosis causing distorted architecture and peri-muscular rosary like lymphoid aggregates are the hallmark features favoring Crohn's disease. In our case mucosa showed superficial ulceration and acute inflammation with maintained crypt architecture. Fibrosis was restricted to submucosa causing thickening of plicae semilunaris which intern caused luminal narrowing and stricture [7]. Other hall mark features of IBD/Crohn's disease as described above was not seen in our case.

Abdominal Tuberculosis is still a common condition in this part of the world, with patients regularly presenting with features of intestinal obstruction. Mycobacterium tuberculosis effects the intestine, peritoneum, mesentery, omentum and intrabdominal organs like liver and spleen. Intestinal tuberculosis does present with strictures, predominantly in the ileocaecal region, with features of intestinal obstruction. However, this is usually supported by a poor immune response, radiological features of pulmonary Koch's, loss of weight and appetite, with bloody diarrhea and night sweats [7]. Morphologically, there is evidence of enlarged mesenteric nodes demonstrating caseating necrosis, along with chronic granulomatous lesions with presence of military like tubercles along the mesentery and omentum. The strictures typically show multiple transverse ulcers in the ileocaecal region with granulomas [7].

Other pathological tests for Tuberculosis and Crohn's disease which were done, ruled both out. The patient also gave no history of NSAID usage, but with pathological findings correlating with the same, which indicates a rare occurrence of Diaphragm disease in the absence of NSAID usage.

Diagnosing diaphragm disease is difficult as it presents with generalised symptoms of bowel obstruction. The use of capsule endoscopies to aid the diagnosis of this condition has been reported. But it runs risk of retention of the capsule when the lumen is significantly narrow, hence it's not recommended if patient presents with symptoms of obstruction [4]. In certain cases of diaphragm disease, CT scan has been reported to not show any specific abnormalities as it lacks the resolution required to highlight the diaphragm [8]. But in our case, there were visible findings of strictures which were noted in the small bowel.

The current treatment of choice involves surgical resection of the stricture segment. A stricturoplasty may be done in cases of solitary or recurrent strictures [9]. Use of other methods of treatment like double balloon enteroscopy and dilation have been reported, without substantial evidence of its effectiveness [10]. However in our case the involved bowel segment had multiple strictures and hence the decision to resect and anastomose was taken.

Studies have shown the active correlation of NSAID and the development of strictures leading to diaphragm disease, thereby prompting the need to withhold this liable drug with evidence of absence of further recurrence [9]. However, once the patient has presented with features of intestinal obstruction, intervention either in the form of surgical or minimal invasive techniques should be employed to alleviate the symptoms and obtain a tissue biopsy to reach this diagnosis as there are subtle microscopic findings which help in differentiating it from common etiological causes of strictures like Crohn's disease and Tuberculosis. Abstaining from NSAIDs in previously diagnosed diaphragm disease patients and the absence of recurrence, thereby prompted us to avoid the same in our patient. Although our patient gave no history of NSAID usage, the strong association between this drug and the formation of strictures, did influence us to take this decision.

Our patient vehemently refutes any use of NSAID drugs, rendering this an exceptional case of Diaphragm disease in the absence of these culpable agents. There is only limited literature evidence of such an occurrence, and we present the successful treatment of this unique case. There are no published guidelines shedding light on the pathophysiological changes of idiopathic diaphragm disease in the absence of NSAID use. Therefore, in patients exhibiting symptoms of intermittent bowel obstruction, healthcare providers should consider diaphragm disease as a potential differential diagnosis to prevent misdiagnosis and inappropriate management.

## 8 Conclusion

This unique case of idiopathic diaphragm disease without NSAID exposure emphasizes the importance of considering alternative causes in patients with recurrent bowel obstructions. While this case underscores the rare occurrence of diaphragm disease without NSAID involvement, further research is needed to understand the underlying mechanisms. Healthcare professionals should maintain a high index of suspicion for diaphragm disease, even in the absence of NSAID history, to ensure accurate diagnosis and appropriate management of patients presenting with intermittent bowel obstruction.

## Informed consent

Written informed consent was obtained from the patient for publication of this case report and accompanying images. A copy of the written consent is available for review by the Editor-in-Chief of this journal on request.

## Provenance and peer review

Not commissioned, externally peer-reviewed.

## Ethical approval

Ethical approval was received from the Institutional Ethics Committee of Kasturba Medical College, Mangalore (Reg. no. ECR/541/Inst/KA/2014/RR-17).

## Funding

No sources of funding.

## Author contribution

Dr. Rajesh R Ballal – Reviewing and Editing the article, Surgical operation

Dr. Talha Ahmed – Study concept, Study design, Data collection, Writing of original draft, Surgical Operation

Dr. Divya Achutha Ail - Study concept, writing and editing of article

Dr. Sumith Marian Colaco– Study concept, writing and editing of article

## Guarantor

Dr. Rajesh R Ballal

## Research registration number

N/A.

## Conflict of interest statement

The authors have NO affiliations with or involvement in any organization or entity with any financial interest (such as honoraria; educational grants; participation in speakers' bureaus; membership, employment, consultancies, stock ownership, or other equity interest; and expert testimony or patent-licensing arrangements), or non-financial interest (such as personal or professional relationships, affiliations, knowledge or beliefs) in the subject matter or materials discussed in this manuscript.
